# Masking release in cortical auditory evoked potentials with speech stimulus

**DOI:** 10.1590/2317-1782/20212020334en

**Published:** 2022-12-19

**Authors:** Mônyka Ferreira Borges Rocha, Denise Costa Menezes, Danielle Samara Bandeira Duarte, Silvana Maria Sobral Griz, Ana Claudia Figueiredo Frizzo, Pedro de Lemos Menezes, Cleide Fernandes Teixeira, Karina Paes Advíncula

**Affiliations:** 1 Programa de Pós-graduação em Saúde da Comunicação Humana, Universidade Federal de Pernambuco - UFPE - Recife (PE), Brasil.; 2 Programa de Pós-graduação em Saúde da Comunicação Humana, Departamento de Fonoaudiologia, Universidade Federal de Pernambuco - UFPE - Recife (PE), Brasil.; 3 Departamento de Fonoaudiologia, Universidade Federal de Pernambuco - UFPE - Recife (PE), Brasil.; 4 Programa de Pós-graduação em Fonoaudiologia, Universidade Estadual Paulista Julio de Mesquita Filho - (UNESP) - São Paulo (SP), Brasil.; 5 Departamento de Fonoaudiologia, Universidade Estadual de Ciências da Saúde de Alagoas - UNCISAL - Maceió (AL), Brasil.

**Keywords:** Electrophysiology, Evoked Potentials Auditory, Speech Perception, Perceptual Masking, Hearing

## Abstract

**Purpose:**

To analyze the effect of masking on the Cortical Auditory Evoked Potential with speech stimulus in young adults.

**Methods:**

Fourteen individuals aged between 19 and 28 years of both sexes with no hearing loss participated in the study. The Cortical Auditory Evoked Potential examination was performed with synthetic speech stimulus /*ba*/ simultaneous to Speech Shaped Noise presented under three conditions: steady noise with a 30 dB SPLep intensity (weak steady noise), steady noise with a 65 dB SPLep intensity o (strong steady noise) and modulated noise with 30 dB SPLep and 65 dB SPLep intensities at 25Hz and modulation period of 40 ms.

**Results:**

Higher latencies were observed in the cortical components, except P2, in the condition of strong steady noise and more meaningful measures of amplitude of the cortical components P1, N1 and P2 in the condition of modulated noise with statistically significant difference in comparison to the strong steady noise condition. There was worse wave morphology in the condition of strong steady noise, when compared to the other records. The average electrophysiological thresholds for the conditions of strong steady noise and modulated noise were 60 dB SPLep and 49 dB SPLep, respectively, showing a 11.7 dB mean difference.

**Conclusion:**

We could infer that there was a lower masking effect of modulated noise when compared to the strong steady noise condition, in the amplitude measurements of the cortical components and an average difference of 11.7 dB between the electrophysiological thresholds (interpreted as the measure of the Masking Release).

## INTRODUCTION

In common social situations of hearing, the listener faces speech conditions simultaneous to noise, causing message distortion or fragmentation, as a result of the masking caused by the competitive noise^(1)^.

Despite the masking caused by the background noise, individuals with normal auditory functions are able to recognize speech signals in the presence of competitive noise through acoustic fluctuations in the temporal envelopes of sound and noise signals^(2)^. These temporal oscillations of noise might occur in intensity of frequency spectrum, generating a better perception of the speech acoustic clues, when compared to situations in which the background noise is continuous^(3)^.

The speech recognition effect caused by available signs of the target stimulus/speech when facing masking fluctuations is called masking release, which is translated into Portuguese as ‘*Benefício do Mascaramento Modulado* - BMM’^(4)^.

Studies on the BMM phenomenon evidenced that physical features of the masking noise are directly related to its magnitude^(5)^, such as the modulation rate, pointing out that lower rates generate larger temporal spaces of lower amplitude that favor the speech perception^(6)^.

The threshold of detection of a signal in the presence of a modulated masking is usually considered lower than that in a constant/steady masking. A behavioral study aiming to determine the BMM magnitude reported an improvement of 15 to 25 dB in the speech recognition threshold with the noise modulation rate between 8 and 20 Hz^(7)^.

Regarding electrophysiological measurements, the threshold difference between the two masking conditions is taken as a measure representing the individuals’ temporal resolution ability^(8)^.

Despite the existence of BMM studies using psychoacoustic measurements in a sample of Brazilian individuals who are native speakers of Portuguese^(4)^, the behavior of the cortical potentials in response to this phenomenon is still unknown, and there are no normality parameters for this normal-hearing population.

Considering the modulated noise temporal fluctuations, we assume that the electrophysiological responses of the Cortical Auditory Evoked Pontentials (PEAC, Portuguese acronym) undergo some modification regarding their latency, amplitude, and electrophysiological threshold, thus generating interference in the temporal processing.

Recognizing the importance of knowing about the behavior of cortical responses to BMM, we consider the study on PEAC with speech stimulus in normal-hearing individuals indispensable. It enables the improvement of diagnosis tests, electrophysiological markers for the auditory processing abilities, and treatment planning to favor speech understanding in noisy situations.

For this reason, this study is characterized as a pioneer for developing an investigation that has not been carried out so far in native speakers of Brazilian Portuguese using more accurate acquisition parameters in the electrophysiological threshold research. Thus, this study aims to analyze the effect of steady and modulated masking on the cortical auditory evoked potential with speech stimulus in young adults.

## METHODS

This research protocol is based on the Resolution nº 466/2012 by the Conselho Nacional de Saúde - CNS (Brazilian National Health Council) for studies on human beings, and was approved by the Ethics Committee for Research on Human Beings of the Federal University of Pernambuco (UFPE), with technical opinion n° 3.555.712.

This is an analytical, observational, and transversal study developed in the Audiology Laboratory of the Phono-Audiology Department at UFPE in the period between October 2019 and April 2020.

The inclusion criteria required individuals between 18 and 28 years old, without hearing loss, while individuals with neurological and/or psychiatric history, cognitive deficits, or malformation of the auricular pavilion and external acoustic meatus, which could prevent the Auditory Evoked Potential examination, were excluded.

The sample included 14 young adults, and the convenience non-probabilistic sampling was used. The participants were recruited after dissemination of the research using electronic media, and all over the university campus.

All participants were instructed regarding the collection objectives and procedures, and after having accepted to take part in the research, they signed two copies of the Free and Informed Consent Form - (TCLE, Brazilian acronym). Next, the pre-collection exams were scheduled, and the research eligibility criteria were verified.

### Pre-collection exams

The researchers carried out a thorough anamnesis of the individuals’ health, basic hearing tests (inspection of the external acoustic meatus, audiometry, and imitanciometry) and the Montreal Cognitive Assessment test^(9)^.

The existence of alterations in the external and/or medium ear was investigated by inspecting the external acoustic meatus, in addition to the imitanciometry using a 226 Hz probe to obtain the static complacency results and investigate the acoustic reflexes. The presence of a type A tympanometric curve^(10)^ and the presence of ipsilateral and contralateral reflexes were considered normality^(11)^. The audiometry examination provided the thresholds for frequencies between 250 Hz and 8000 Hz, including the 3000 Hz and 6000 Hz inter-octaves of both ears, using supra-aural earphones in an acoustic cabin. As normality standard, we considered the presence of auditory thresholds with average below 20 dB NA in the frequencies 500 Hz, 1000 Hz, 2000 Hz, and 4000 Hz^(12)^. When applying the MoCA, we considered the score equal to or over 26 points described in the test as a normality result.

### Acquisition of cortical auditory evoked potentials

#### a) Stimuli

A synthetic speech /ba/ stimulus and a *Speech Shaped Noise* (SSN) were used^(13)^. The /ba/ stimulus was presented as a modified wave for a 24.414 Hz rate to be compatible to the digital signal of the *Tucker-Davis Technologies* (TDT- RZ6) processing platform, and calibrated in reference to the dB SPLep of a steady 1kHz tone, equivalent peak (dB SPLep). The SSN masking noise presented a multilingual speech spectrum and was elaborated at the Laboratory of Speech and Hearing Sciences of the University of North Carolina at Chapel Hill, in the USA.

#### b) Procedure

The individuals that were eligible to the study underwent the PEAC test with the Intelligent Hearing Systems - HIS equipment. The speech stimulus /ba/ and the noise were presented in a monoaural way to the right ear via electromagnetically shielded insertion phone (ER2), directly linked from the TDT- RZ6 to the individual. Disposable ear buds were used for each participant.

To register the potentials, a recording system was synchronized between the Smart EP of the IHS with the TDT- RZ6 through a time-event marker ("Trigger") coincident to the start of each /ba/ stimulus. A synthesis of the cortical potential register parameters is presented below (Chart 1).

**Chart 1:** Synthesis of parameters for the register of Cortical Auditory Evoked Potential

**Smart EP**	**Intelligent Hearing Systems - IHS**
Model	Opti-Amp 8008 model
Synchronization	Tucker-Davis Technologies (TDT- RZ6)
Marker	Time-event (Trigger)
**Stimulus**	
Speech stimulus	/ba/
Duration	80ms
Intensity	65 dB SPLep
Presentation rate	3.8 e/s
**Noise**	
Speech noise	Speech Shaped Noise (SSN)
Duration	100ms
Onset/offset slopes	10ms
Weak steady noise	30 dB SPLep
Strong steady noise	65 dB SPLep
Modulated noise	25 Hz and 30 and 65 dB SPLep intensities
Modulation period	40ms
**Acquisition parameters**	
Window	512ms
Filters	1 and 30 Hz
Impedance	≤ 5 kΩ

**Caption:** ms = milliseconds

The participant was positioned in a recliner chair within the acoustic cabin and watched a video without audio during the exam. The participants were asked not to sleep during the evaluation. Their skin was cleaned with alcohol 70% and abrasive gel, brand NuPrep® before the electrodes were placed in the following configurations: two reference electrodes of negative polarity positioned in the region of the right (A1) and left (A2) lobes; one electrode of positive polarity placed on the vertex (Cz), and the ground electrode placed on the forehead lower region (Fpz).

**Figure 1:**
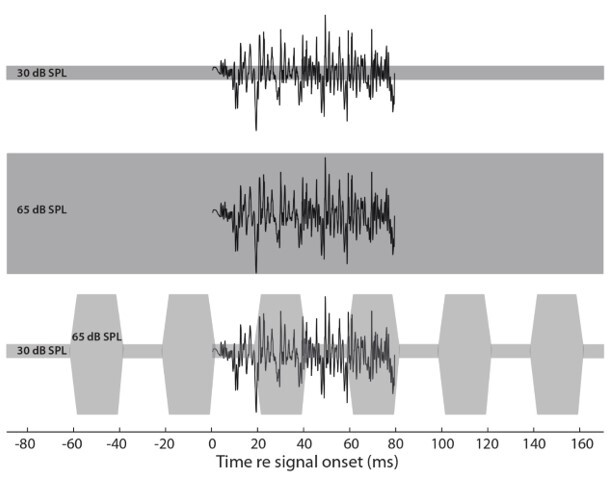
Illustration of the /ba/ stimulus in the three noise conditions. Cortical waves during weak steady noise (A); cortical waves during strong steady noise (B); cortical waves during modulated noise (C)

To obtain the PEAC, noise was presented simultaneous to the /ba/ stimulus in three conditions (Figure 1): a) /ba/ and steady noise with a 30 dB SPLep intensity (weak steady noise); b) /ba/ and steady noise with a 65 dB SPLep intensity (strong steady noise); c) /ba/ and modulated noise at 25 Hz with 30 and 65 dB SPLep intensities. The modulation period was used to allow the /ba/ stimulus appearance between the intensity changes, aiming to observe the BMM.

The presentation of different noise conditions was carried out randomly in each individual’s examination. The participants’ electrophysiological threshold was investigated in the strong steady noise and modulated noise conditions by decreasing gradually the intensity of the speech stimulus in 10 to 10 dB up to the disappearance of the P1-N1-P2 complex, and then decreasing it in 2 to 2 dB up to its appearance. Each exam lasted around an hour.

#### a) Trace analysis

Latency measurements (in milliseconds - ms) and amplitude (in microvolts - µV), as well as the morphology of P1, N1, and P2 waves were analyzed regarding the three noise presentation conditions, assessing the difference between these responses.

The trace register was identified by measuring the latency and amplitude of the P1, N1, and P2 cortical analysis that were analyzed by three blinded researchers, who were experienced in electrophysiology and agreed with the identification and marking of the potentials. The P1 component was considered as the first most robust positive cortical wave around 50ms, the N1 component was analyzed as the valley subsequent to the P1 wave, with higher negativity, and the P2 response was identified as the most robust positive wave after N1.

By obtaining the electrophysiological threshold in the strong steady noise and modulated noise conditions, it was possible to measure each individual’s BMM value upon the difference in decibels (dB SPLep) in both noise presentation situations.

### Data analysis

The statistical analysis was carried out using the *Statistical Package for the Social Sciences* (SPSS) program, version 20.0. The results were expressed through statistical measures of median and interquartile distance. To describe such measures, graphs and tables were designed. The normality of the samples was verified using the Shapiro-Wilk test, and an abnormal distribution was observed. To obtain the significant difference of averages between the cortical components in each noise condition, and to carry out a comparison between the electrophysiological thresholds, the Wilcoxon test for paired data was employed. The difference was considered significant when the p-value <0.05.

## RESULTS

Out of the 14 research participants, nine (64.28%) were women and six (35.72%) were men, their age ranged between 19 and 28 years old (mean 23 years ± 2.81), and prevalence of right brain dominance was observed, with 13 right-handed individuals (92.8%). Regarding schooling data, seven (50%) had already concluded higher education, one participant had concluded high school, and six were undergraduate students.

When describing the latency values of the cortical components in the different noise conditions in the presence of the /ba/ stimulus (Table 1), lower latency value was observed in the weak steady noise condition, while in the strong steady noise condition, higher latency value was observed, except for the P2 component.

**Table 1:** Comparison of latency averages of the P1, N1, and P2 components between the different noise conditions in a sample containing 14 individuals

Latency	Weak steady noise	Strong steady noise	Modulated noise	Wilcoxon
(ms)	M_d_	M_d_	M_d_	p-value
	(Q_25_ - Q_75_)	(Q_25_ - Q_75_)	(Q_25_ - Q_75_)	
P1 Component	53.0	77.5	73.5	p 0.004 ^a^
	(44.25 - 57.25)	(57.2 - 105.2)	(60.0 - 88.7)	p 0.003 ^b^
				p 0.510 ^c^
N1 Component	111.0	137.5	137.5	p 0.009 ^a^
	(102.0 - 118.0)	(124.0 - 187.5)	(129.7 - 153.2)	p 0.002 ^b^
				p 0.730 ^c^
P2 Component	171.5	199.0	208.5	p 0.039 ^a^
	(159.0 - 188.0)	(166.5 - 227.0)	(202.2 - 210.7)	p 0.001 ^b^
				p 0.116 ^c^

**Caption:** ms = milliseconds; M_d_ = median; (Q_25_, Q_75_) = interquartile distance

a statistically significant difference of averages between the weak and strong steady noises;

b statistically significant difference of averages between the weak steady and the modulated noises;

c absence of statistical significance of the averages between strong steady and modulated noises

In the comparison between the latency averages of the cortical components between the noise conditions, statistically significant difference was observed between the two steady noise conditions, as well as between the weak steady noise and the modulated noise.

When describing the values of amplitudes of the cortical components in the different noise conditions (Table 2), lower amplitude was observed in the strong steady noise condition, while the modulated noise and the weak steady noise groups obtained higher amplitude, without statistically significant difference.

**Table 2:** Comparison between amplitude averages of the P1, N1, and P2 components in different noise conditions in a sample containing 14 individuals

**Amplitude**	**Weak steady noise**	**Strong steady noise**	**Modulated noise**	**Wilcoxon**
**(µV)**	**M_d_**	**M_d_**	**M_d_**	**p-value**
	**(Q_25_ - Q_75_)**	**(Q_25_ - Q_75_)**	**(Q_25_ - Q_75_)**	
P1 Component	5.5	4.2	5.7	p 0.005 ^a^
	(4.3 - 6.6)	(3.0 - 4.6)	(4.7 - 6.5)	p 0.009 ^b^
				p 0.510 ^c^
N1 Component	4.6	1.6	3.6	p 0.001 ^a^
	(4.0 - 6.7)	(0.7 - 2.6)	(2.8 - 5.4)	p 0.004 ^a^
				p 0.140 ^a^
P2 Component	4.4	1.5	4.5	p 0.001 ^a^
	(2.6 - 6.8)	(0.8 - 2.3)	(3.2 - 5.9)	p 0.001 ^a^
				p 0.900 ^a^

**Caption:** µV = microvolts; M_d_ = median; (Q_25_ - Q_75_) = interquartile distance

a statistically significant difference of averages between strong and weak steady noises;

b statistically significant difference of averages between the strong steady and modulated noises;

c absence of statistical significance between the weak steady and modulated noises

However, when comparing the amplitude averages, statistically significant difference was observed between the strong steady noise and the modulated noise groups, as well as between the two types of steady noises (Table 2).

**Figure 2:**
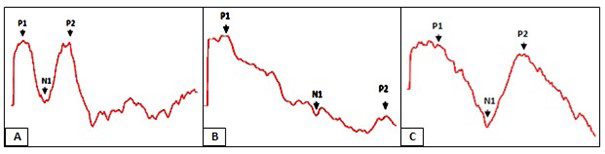
Illustration of the morphology of cortical waves in the three noise conditions

As for the morphology of the waves registered in the different noise situations, poorer wave presentation was observed in the strong steady noise condition when compared to the other registers (Figure 2A, B, C).

In the electrophysiological threshold investigation, two individuals did not take part in the register because they missed the examination due to personal reasons, therefore, 12 participants were examined. We could observe that the threshold was lower in the modulated noise condition (Table 3).

**Table 3:** Description of median and interquartile distance of the thresholds of strong steady noise, modulated noise, and BMM, in a sample containing 12 individuals

**Variables**	**Mean**	**Median**	Q_25_ - Q_75_
Strong steady noise threshold - dB SPLep	60.9	60.0	57.7 - 65.0
Modulated noise threshold - dB SPLep	49.1	49.0	45.0 - 55.0
BMM - dB SPLep	11.7	12.0	6.2 - 15.0

**Caption:** dB SPLep = decibel sound pressure level; BMM = (Benefício do Mascaramento Modulado) Masking release; Q_25_ - Q_75_ = interquartile distance. Black spectrum = speech stimulus; Gray band (30 dB) = weak steady noise; Gray band (65 dB) = strong steady noise; Fragmented gray band (30, 65 dB) = modulated noise; dB SPL (Decibel - Sound Pressure Level)

**Figure 3:**
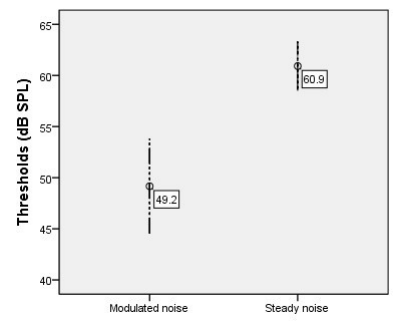
Electrophysiological threshold for the /ba/ stimulus as a function of the type of noise

We could also observe that the threshold in the strong steady noise condition was markedly high when compared to the modulated noise condition (Figure 3), with statistical significance in the Wilcoxon test, p=0.003. Regarding the BMM, we could see that the confidence interval ranged from 7.7 to 15.7. In addition, the mean difference between the electrophysiological thresholds resulted in a 11.7 dB BMM.

## DISCUSSION

The complex of the P1, N1, and P2 cortical waves when evoked by a speech stimulus reveals exogenous responses referring to the acoustic features of the sound processing^(14)^. In this study, we could observe the presence of the cortical complex in all individuals evaluated, which suggests that the speech stimulus was properly received at the level of the auditory cortex.

In this study, the BMM investigation process considering cortical auditory potentials is related to the detection of a speech stimulus at a central level, reflecting postsynaptic excitatory activity at the level of the thalamus and primary auditory cortex, in addition to the association areas^(15)^.

The analysis of latencies and amplitudes of the cortical components in the processing of these complex signals allows the researchers to infer about the influence of time in the stimulus perception and about the magnitude of the cortical activity, respectively^(15)^.

This study revealed that longer time of stimulus detection occurred in the condition in which the /ba/ was presented with the strong steady noise, evidenced by higher latencies observed in the cortical components, except for P2 (Table 1). This fact was due to the higher masking effect caused by the strong steady noise on the latencies, with a significant difference when compared to the weak steady noise condition (which presented lower latencies); however, without statistical difference when compared to the modulated noise, in which the masking effect was similar.

Similar results of increased latency in cortical potentials evoked by speed stimulus in the presence of steady noise were observed in young adults, resulting in progressively delayed latencies of the P1, N1, and P2 components in this noise condition ^(16)^. Such latency delay might be ascribed to the masking effect in the synchronization of the neural activity subjacent to the auditory processing, since noise alters the auditory system perception time.

Regarding the amplitude values, as a response representing the magnitude of the cortical activity, we could observe lower amplitude, that is, lower response magnitude of the P1, N1, and P2 components, when the /ba/ stimulus was presented with strong steady noise. However, higher latencies were observed with the modulated noise and weak steady noise, with significant difference when compared to the strong steady noise condition (Table 1).

The masking effect caused by the modulated noise on the amplitude values was lower than that caused by the strong steady noise (Table 2). Therefore, we considered better magnitude of the cortical activity in the verbal stimulus processing in the modulated noise situation.

This result might be explained by the fact that noise intensity modulations cause reduction in the signal-noise relation, and consequently, increase the amplitude of the evoked stimulus^(5)^. Thus, the stimulus amplitude increase with the noise modulations results in decreased latency and increased magnitude of the auditory system responses, since these measures vary inversely and directly with the stimulus amplitude, respectively^(17)^.

When comparing the cortical potentials evoked by the monaural /ba/ stimulus in the steady noise situation produced by the speech and the modulated wide band noise, at a 65 dB SPLep fixed signal level and different types of signal-noise relations, other researchers also observed a systematic decrease in the amplitude and increase in latency in the steady noise condition ^(18)^.

Considering the morphology of the P1-N1-P2 complex in the different noise conditions, the worst wave configuration was observed with the strong steady noise when compared to the other registers. It might be explained by the greater interference observed in the latency and amplitude measures in this condition.

Some studies have also reported robust cortical potentials with modulated noise, when compared to the steady noise, indicating lower masking effect of the sound signal in the modulated noise^(16,19)^.

When investigating the electrophysiological threshold in this study, we observed a lower threshold with the modulated noise, and statistically significant difference in relation to the strong steady noise threshold (Table 3; Figure 3).

The highest threshold observed in the strong steady noise condition might indicate that the temporal masking effects were more robust in that condition.

Therefore, a 11.7 dB lower average threshold occurred with the modulated noise, which agreed with the literature that reports that the signal detection threshold in the presence of a modulated masking is usually weaker than that occurring in the presence of constant/steady masking^(20)^.

This threshold difference between the two masking conditions might be taken as a measure representing the individuals’ ability of temporal resolution, being related to the integrity of the temporal processing^(8)^.

Regarding the 25 Hz noise modulation rate of this study, some studies have reported that lower modulation rates, for example, between 8 Hz and 20 Hz, produce longer temporal spaces of lower noise amplitude and, consequently, generate a better BMM magnitude^(5,6)^.

When investigating the BMM in the PEAC with speech stimulus and relate them to behavioral measures, average electrophysiological thresholds of approximately 69 dB with steady noise, and 55.5 dB with modulated noise were obtained, reaching a mean threshold of approximately 13.5 dB lower with the modulated noise, close to that found in this study. Taking that into consideration, those authors pointed out that the speech electrophysiological threshold was lower in the modulated masking condition when compared to the steady masking and associated the masking release to the individuals’ temporal processing ability^(8)^.

In studies on behavioral measures, in which the BMM magnitude was investigated in normal hearing individuals, a variation between 15 and 25 dB improvement in the speech recognition was observed with the masking noise modulation noise between 8 and 20 Hz^(7)^.

Another study that observed the similarity of the BMM magnitude between the electrophysiological and behavioral domains reported that the electrophysiological tests are not only informative of the underlying mechanisms, but that they can also evaluate temporal processing abilities^(8)^. It also reported that the PEAC thresholds evoked by verbal stimulus might be reliable predictors of speech detection thresholds in steady and modulated masking situations.

In this study, the smaller difference between the electrophysiological thresholds, that is, the lowest BMM found was zero dB, no negative result of this phenomenon was found in the individuals analyzed. The literature reports that BMM favors the brain in the processing of speech acoustic clues that do not coincide with the masking noise features^(2)^.

These findings are predominantly limited to young adults without hearing loss; however, they can be used to support studies on other age groups and populations with specific alterations of the hearing abilities, thus contributing to the research on BMM in PEAC. The BMM analysis should be carried out in the young, adult, and older population, enabling the assessment of temporal resolution ability in the presence of the masking phenomenon.

## CONCLUSION

The findings of this study demonstrated a lower masking effect of the modulated noise in the amplitude measures of the P1, N1, and P2 cortical components, which might indicate BMM signals. A 11.7 dB BMM represented by the difference between the average electrophysiological thresholds suggests lower interference of the temporal masking in the condition in which the /ba/ stimulus is presented with modulated noise. According to these results and the contribution to the BMM in PEAC research, we consider the use of modulated noise as the most efficient masking in this evaluation.
