# Supplementary material for: Masking release in cortical auditory evoked potentials with speech stimulus
Source: Codas. 2022 Dec 19;35(1):e20200334. doi: 10.1590/2317-1782/20212020334en (PMC10010424; doi:10.1590/2317-1782/20212020334en)
Supplement: Translation

# Translation: O benefício do mascaramento modulado nos potenciais evocados auditivos corticais com estímulo de fala

**DOI:** 10.1590/2317-1782/20212020334pt

**Authors:** Mônyka Ferreira Borges Rocha, Denise Costa Menezes, Danielle Samara Bandeira Duarte, Silvana Maria Sobral Griz, Ana Claudia Figueiredo Frizzo, Pedro de Lemos Menezes, Cleide Fernandes Teixeira, Karina Paes Advíncula

**Affiliations:** 1 Programa de Pós-graduação em Saúde da Comunicação Humana, Universidade Federal de Pernambuco - UFPE - Recife (PE), Brasil.; 2 Programa de Pós-graduação em Saúde da Comunicação Humana, Departamento de Fonoaudiologia, Universidade Federal de Pernambuco - UFPE - Recife (PE), Brasil.; 3 Departamento de Fonoaudiologia, Universidade Federal de Pernambuco - UFPE - Recife (PE), Brasil.; 4 Programa de Pós-graduação em Fonoaudiologia, Universidade Estadual Paulista Julio de Mesquita Filho - (UNESP) - São Paulo (SP), Brasil.; 5 Departamento de Fonoaudiologia, Universidade Estadual de Ciências da Saúde de Alagoas - UNCISAL - Maceió (AL), Brasil.

**Keywords:** Eletrofisiologia, Potenciais Evocados Auditivos, Percepção da Fala, Mascaramento Perceptivo, Audição

## Abstract

**Objetivo:**

analisar o efeito do mascaramento estável e modulado no Potencial Evocado Auditivo Cortical com estímulo de fala em adultos-jovens.

**Método:**

participaram 14 indivíduos com idades entre 19 e 28 anos de ambos os sexos e sem perda auditiva. O exame de Potencial Evocado Auditivo Cortical foi realizado com estímulo de fala sintética /ba/ simultâneo ao ruído Speech Shaped Noise apresentado em três condições: ruído estável com intensidade de 30 dB NPSpe (ruído estável fraco), ruído estável com intensidade de 65 dB NPSpe (ruído estável forte) e ruído modulado em intensidade de 30 dB NPSpe e 65 dB NPSpe em 25Hz e com período de modulação de 40 ms.

**Resultados:**

foram observadas maiores latências nos componentes corticais, exceto P2, na condição de ruído estável forte e medidas mais robustas de amplitude dos componentes corticais P1, N1 e P2 na condição de ruído modulado com diferença estatística significativa na comparação com a condição de ruído estável forte. Houve pior morfologia na condição de ruído estável forte, quando comparado aos demais registros. Os limiares eletrofisiológicos médios para as condições de ruído estável forte e ruído modulado foram 60 dB NPSpe e 49 dB NPSpe, respectivamente, mostrando 11,7 dB de diferença média.

**Conclusão:**

podemos inferir que houve um menor efeito mascarante do ruído modulado, comparado à condição de ruído estável forte, nas medidas de amplitude dos componentes corticais e uma diferença média de 11,7 dB entre os limiares eletrofisiológicos (interpretado como a medida do Benefício do Mascaramento Modulado).

## INTRODUÇÃO

Em situações sociais comuns de escuta, o ouvinte se depara com condições que a fala ocorre concomitante com o ruído, causando distorções ou fragmentações na mensagem, resultante do mascaramento causado pelo ruído competitivo^(1)^.

Apesar do mascaramento causado pelo ruído de fundo, indivíduos com audição normal são capazes de reconhecer sinais de fala na presença de sons competitivos por meio de flutuações acústicas nos envelopes temporais de sinais sonoros e ruídos^(2)^. Tais oscilações temporais do ruído podem acontecer em intensidade ou espectro de frequência, gerando uma melhor percepção das pistas acústicas de fala, quando comparado em situações que o ruído de fundo é contínuo^(3)^.

O efeito no reconhecimento de fala causado por sinais disponíveis do estímulo alvo/fala diante das flutuações de mascaramento é denominado *masking release*, e traduzido para português como Benefício do Mascaramento Modulado - BMM^(4)^.

Estudos sobre o fenômeno do BMM evidenciam que características físicas do ruído mascarante estão diretamente relacionadas à sua magnitude^(5)^, como a taxa de modulação, apontando ainda que taxas mais baixas geram maiores espaços temporais de menor amplitude e que facilitam a percepção da fala^(6)^.

Considera-se que o limiar de detecção de um sinal em presença de um mascaramento modulado é geralmente mais baixo do que em mascaramento constante/estável. Em estudo comportamental para determinar a magnitude do BMM constatou-se uma melhora de 15 a 25 dB no limiar de reconhecimento de fala com taxa de modulação do ruído entre 8 e 20 Hz^(7)^.

Para medidas eletrofisiológicas, a diferença no limiar entre as duas condições de mascaramento é tomada como uma medida representante da habilidade de resolução temporal dos indivíduos^(8)^.

Apesar de haver estudos do BMM por meio de medidas psicoacústicas em indivíduos de nacionalidade brasileira e falante nativos do português^(4)^, ainda não se conhece o comportamento dos potenciais corticais diante deste fenômeno, não havendo parâmetros de normalidade para esta população normouvinte.

Diante das flutuações temporais do ruído modulado, presume-se que as respostas eletrofisiológicas dos Potenciais Evocados Auditivos Corticais (PEAC) sofram modificações quanto a sua latência, amplitude e limiar eletrofisiológico, gerando interferência no processamento temporal.

Considerando a importância do conhecimento sobre o comportamento das respostas corticais diante do BMM, torna-se indispensável o estudo dos PEAC com estímulo de fala em indivíduos normouvintes, a fim de permitir o aprimoramento dos testes de diagnóstico, marcadores eletrofisiológicos para habilidades do processamento auditivo e planejamento terapêutico que facilitem a compreensão da fala em situações de ruído.

Por este motivo, este estudo caracteriza-se como pioneiro por se tratar de uma investigação ainda não realizada em nativos de língua portuguesa, utilizando parâmetros de aquisição mais precisos na pesquisa dos limiares eletrofisiológicos. Diante disso, o presente estudo tem como objetivo analisar o efeito do mascaramento estável e modulado no Potencial Evocado Auditivo Cortical com estímulo de fala em adultos jovens.

## MÉTODO

O protocolo desta pesquisa é baseado na Resolução nº 466/2012 do Conselho Nacional de Saúde - CNS para estudos com seres humanos e foi aprovado pelo Comitê de Ética e Pesquisa em Seres Humanos da Universidade Federal de Pernambuco (UFPE), com o parecer de número 3.555.712.

A pesquisa trata-se de um estudo analítico, do tipo observacional e transversal, realizado no Laboratório de Audiologia do Departamento de Fonoaudiologia da UFPE no período entre outubro de 2019 e abril de 2020.

Foram estabelecidos como critérios de inclusão da pesquisa, indivíduos entre 18 e 28 anos de idade sem perda auditiva, sendo excluídos indivíduos com história de doenças neurológicas e/ou psiquiátricas, déficits cognitivos, indivíduos com malformações de pavilhão auricular e meato acústico externo que impossibilite a realização do exame do Potencial Evocado Auditivo.

Participaram do estudo 14 indivíduos jovens-adultos e a amostragem foi do tipo não probabilística por conveniência. Os participantes foram recrutados após divulgação da pesquisa por meios eletrônicos e por todo o campus universitário.

Todos os participantes receberam orientações quanto aos objetivos e procedimentos da coleta e após aceitarem participar da pesquisa, assinaram ao Termo de Consentimento Livre e Esclarecido - TCLE em duas vias. Em seguida, foram agendados os exames de pré-coleta para resposta aos critérios de elegibilidade da pesquisa.

### Exames de pré-coleta

Os pesquisadores realizaram em data agendada, anamnese detalhada a respeito da saúde do indivíduo, exames audiológicos básicos (inspeção do meato acústico externo, audiometria e imitanciometria) e teste de Avaliação Cognitiva Montreal - MoCA ^(9)^.

A existência de alterações na orelha externa e/ou média foi investigada por meio da inspeção do meato acústico externo, além do exame de imitaniciometria com a utilização da sonda de 226 Hz para aquisição dos resultados de complacência estática e pesquisa dos reflexos acústicos. Como normalidade, foi considerada a presença de curva timpanométrica do tipo A^(10)^ e presença de reflexos ipsilaterais e contralaterais^(11)^. No exame de audiometria foram obtidos os limiares para as frequências entre 250 Hz à 8000 Hz, incluindo as interoitavas de 3000 Hz e 6000 Hz, de ambas as orelhas, através de fones auditivos supra-aurais, em cabina acústica. Como padrão de normalidade foi considerada a presença de limiares auditivos com média inferior a 20 dB NA nas frequências de 500 Hz, 1000 Hz, 2000 Hz e 4000 Hz^(12)^. Na aplicação do MoCA foi considerado como resultado de normalidade a pontuação igual ou superior a 26 pontos, descrita no teste.

### Aquisição dos Potenciais Evocados Auditivos Corticais

#### a) Estímulos

Foram utilizados um estímulo de fala sintética /ba/ e um ruído de fala formatado *Speech Shaped Noise* (SSN)^(13)^. Tal estímulo /ba/ foi apresentado com forma de onda modificada para uma taxa de 24.414 Hz para ser compatível com o sinal digital da plataforma de processamento *Tucker-Davis Technologies* (TDT- RZ6) e calibrado com referência ao dB NPSpe de um tom contínuo de 1kHz, pico equivalente (dB NPSpe). O ruído mascarante SSN possui espectro de fala multilíngue e foi elaborado no Laboratório de Ciências da Audição da Universidade da Carolina do Norte em Chapel Hill, nos Estados Unidos.

#### b) Procedimento

Os indivíduos elegíveis para o estudo realizaram o teste de PEAC com o equipamento Inteligent Hearing Systems - IHS. O estímulo de fala /ba/ e o ruído foram apresentados de forma monoaural para a orelha direita via fone de inserção (ER2) eletromagneticamente blindado, ligados diretamente do TDT- RZ6 ao sujeito. Foram utilizadas olivas descartáveis para cada participante.

Para registro dos potenciais, um sistema de gravação foi sincronizado entre o Smart EP do IHS com o TDT- RZ6 por meio de um marcador de tempo-evento ("Trigger") coincidente com o início de cada estimulo /ba/. Uma síntese dos parâmetros de registro dos potenciais corticais foi demonstrada no Quadro 1.

**Quadro 1:** Síntese dos parâmetros para o registro do Potencial Evocado Auditivo Cortical

**Smart EP**	**Inteligent Hearing Systems - IHS**
Modelo	modelo Opti-Amp 8008
Sincronização	Tucker-Davis Technologies (TDT- RZ6)
Marcador	Tempo-evento (Trigger)
**Estímulo**	
Estímulo de fala	/ba/
Duração	80ms
Intensidade	65 dB NPSpe
Taxa de apresentação	3,8 e/s
**Ruído**	
Ruído de fala	Speech Shaped Noise (SSN)
Duração	100ms
Rampas onset/offset	10ms
Ruído estável fraco	30 dB NPSpe
Ruído estável forte	65 dB NPSpe
Ruído modulado	25 Hz e intensidades de 30 e 65 dB NPSpe
Período de modulação	40ms
**Parâmetros de aquisição**	
Janela	512ms
Filtros	1 e 30 Hz
Impedância	≤ 5 kΩ

**Legenda:** ms = milissegundos

O participante foi posicionado em uma poltrona reclinável dentro de cabina acusticamente tratada e assistindo a um vídeo sem áudio durante a realização do exame. Os participantes foram orientados a não dormir durante a avaliação. A limpeza da pele com álcool 70% e gel abrasivo da marca NuPrep® foi realizada previamente à colocação dos eletrodos nas seguintes configurações: dois eletrodos de referência de polaridade negativa posicionados na região dos lóbulos direito (A1) e esquerdo (A2); um eletrodo de polaridade positiva colocado no vértice (Cz) e o eletrodo terra posicionado na região inferior da fronte (Fpz).

Para aquisição dos PEAC o ruído foi apresentado simultaneamente ao estímulo /ba/ em três condições, como ilustrado na Figura 1: a) /ba/ e ruído estável com intensidade de 30 dB NPSpe (ruído estável fraco); b) /ba/ e ruído estável com intensidade de 65 dB NPSpe (ruído estável forte); c) /ba/ e ruído modulado em 25 Hz nas intensidades de 30 e 65 dB NPSpe. O período de modulação foi utilizado para permitir o aparecimento do estímulo /ba/ entre as mudanças de intensidade, com o objetivo de observar o BMM.

**Figura 1:** Ilustração do estímulo /ba/ nas três condições de ruído. Ondas corticais durante ruído estável fraco (A); ondas corticais durante ruído estável forte (B); ondas corticais durante ruído modulado (C)

A apresentação das diferentes condições de ruído foi realizada de maneira aleatória em cada indivíduo. O limiar eletrofisiológico dos participantes foi pesquisado nas condições de ruído estável forte e ruído modulado, a partir da diminuição da intensidade do estímulo de fala de 10 em 10 dB até o desaparecimento do complexo P1-N1-P2 e em seguida o aumento de 2 em 2 dB até o seu aparecimento. A duração total do exame foi de aproximadamente uma hora em cada participante.

#### a) Análise dos traçados

As medidas de latência (em milissegundos - ms) e amplitude (em microvolts - µV), bem como a morfologia das ondas P1, N1, P2 foram analisadas diante das três condições de apresentação do ruído, avaliando a diferença entre essas respostas.

O registro dos traçados foi identificado com mensuração da latência e amplitude dos componentes corticais P1, N1 e P2, sendo analisados, de maneira cega, por três pesquisadores com experiência em eletrofisiologia que concordaram com a identificação e marcação dos potenciais. O componente P1 foi considerado como a primeira onda cortical positiva mais robusta em torno de 50ms, o componente N1 foi analisado como o vale subsequente à onda P1, com maior negatividade, e a resposta de P2 foi identificada como a onda positiva mais robusta após N1.

Com a obtenção do limiar eletrofisiológico nas condições de ruído estável forte e ruído modulado foi possível mensurar o valor do BMM de cada sujeito diante da diferença em decibéis (dB NPSpe) nas duas situações de apresentação do ruído.

### Análise dos dados

A análise estatística foi realizada por meio do programa *Statistical Package for the Social Sciences* (SPSS) versão 20.0. Os resultados foram expressos através das medidas estatísticas de mediana e distância interquartílica. Para descrição dessas medidas foi utilizada apresentação gráfica e tabular. A normalidade das amostras foi verificada com o teste de Shapiro-Wilk, sendo observada uma distribuição não normal. Para constatar a diferença de médias significativa entre os componentes corticais em cada condição de ruído e para traçar comparação entre os limiares eletrofisiológicos, utilizou-se o teste de Wilcoxon para dados pareados com vinculação entre si. Foi considerada diferença significativa quando p-valor <0,05.

## RESULTADOS

Dos 14 participantes da pesquisa, nove (64,28%) eram do sexo feminino e seis (35,72%) do sexo masculino, com idades variando de 19 a 28 anos (média 23 anos ± 2,81) e prevalência de dominância cerebral direita, com 13 indivíduos destros equivalendo a 92,8%. Quanto ao nível de escolaridade, sete (50%) já havia concluído o ensino superior, um participante possuía o ensino médio completo e seis eram estudantes de graduação.

Na descrição dos valores de latências dos componentes corticais nas diferentes condições de ruído na presença do estímulo /ba/ (Tabela 1), foi observado menor valor de latência na condição do ruído estável fraco, enquanto que na condição de ruído estável forte, observou-se um maior valor de latência, exceto para o componente P2.

**Tabela 1:** Comparação das médias de latência dos componentes P1, N1 e P2 entre as diferentes condições de ruído numa amostra de 14 sujeitos

**Latência**	**Ruído estável fraco**	**Ruído estável forte**	**Ruído modulado**	**Wilcoxon**
(ms)	M_d_	M_d_	M_d_	p-valor
	(Q_25_ - Q_75_)	(Q_25_ - Q_75_)	(Q_25_ - Q_75_)	
Componente				
P1	53	77,5	73,5	p 0,004 ^a^
	(44,25 - 57,25)	(57,2 - 105,2)	(60,0 - 88,7)	p 0,003 ^b^
				p 0,510 ^c^
Componente	111	137,5	137,5	p 0,009 ^a^
N1	(102,0 - 118,0)	(124,0 - 187,5)	(129,7 - 153,2)	p 0,002 ^b^
				p 0,730 ^c^
Componente	171,5	199	208,5	p 0,039 ^a^
P2	(159,0 - 188,0)	(166,5 - 227,0)	(202,2 - 210,7)	p 0,001 ^b^
				p 0,116 ^c^

**Legenda:** ms = milissegundos; M_d_ = mediana; (Q_25_, Q_75_) = distância interquartílica

a diferença estatisticamente significativa das médias entre os ruídos estável fraco e forte;

b diferença estatisticamente significativa das médias entre os ruídos estável fraco e ruído modulado;

c ausência de significância estatística das médias entre os ruídos estável forte e ruído modulado

Na comparação das médias de latências dos componentes corticais entre as condições de ruído foi observada diferença estatisticamente significativa entre as duas condições de ruídos estáveis, como também entre as condições de ruído estável fraco e ruído modulado.

Na descrição dos valores de amplitudes dos componentes corticais nas diferentes condições de ruído (Tabela 2), na condição de ruído estável forte foi observado menor amplitude, enquanto que no grupo de ruído modulado e ruído estável fraco foram obtidas maiores amplitude, sem diferença estatística significativa.

**Tabela 2:** Comparação das médias de amplitude dos componentes P1, N1 e P2 entre as diferentes condições de ruído numa amostra de 14 sujeitos

**Amplitude**	**Ruído estável fraco**	**Ruído estável forte**	**Ruído modulado**	**Wilcoxon**
(µV)	**M_d_**	**M_d_**	**M_d_**	**p-valor**
	**(Q_25_ - Q_75_)**	**(Q_25_ - Q_75_)**	**(Q_25_ - Q_75_)**	
Componente	5,5		5,7	p 0,005 ^a^
P1	(4,3 - 6,6)	4,2	(4,7 - 6,5)	p 0,009 ^b^
		(3,0 - 4,6)		p 0,510 ^c^
Componente	4,6	1,6	3,6	p 0,001 ^a^
N1	(4,0 - 6,7)	(0,7 - 2,6)	(2,8 - 5,4)	p 0,004 ^a^
				p 0,140 ^a^
Componente	4,4	1,5	4,5	p 0,001 ^a^
P2	(2,6 - 6,8)	(0,8 - 2,3)	(3,2 - 5,9)	p 0,001 ^a^
				p 0,900 ^a^

**Legenda:** µV = microvolts; M_d_ = mediana; (Q_25_ - Q_75_) = distância interquartílica

a diferença estatisticamente significativa das médias entre os ruídos estável forte e fraco;

b diferença estatisticamente significativa das médias entre os ruídos estável forte e ruído modulado;

c ausência de significância estatística das médias entre os ruídos estável fraco e ruído modulado

Na comparação das médias de amplitude foi observado diferença estatisticamente significativa entre os ruídos estável forte e ruído modulado, como também entre os dois tipos de ruído estável (Tabela 2).

Quanto à morfologia das ondas registradas nas diferentes situações de ruído, foi possível observar pior apresentação das ondas na condição de ruído estável forte, quando comparado aos demais registros. Figura 2A, B, C.

**Figura 2:** Ilustração da morfologia das ondas corticais nas três condições de ruído

Na pesquisa do limiar eletrofisiológico, dois indivíduos não realizaram o registro pela necessidade de se ausentar do exame por motivos pessoais, permanecendo 12 voluntários. Na Tabela 3, observa-se que o limiar foi menor na condição de ruído modulado.

**Tabela 3:** Descrição da mediana, e distância interquartílica dos limiares de ruído estável forte, ruído modulado e BMM, numa amostra de 12 sujeitos

**Variáveis**	**Média**	**Mediana**	Q_25_ - Q_75_
Limiar de ruído estável forte - dB NPSpe	60,9	60,0	57,7 - 65,0
Limiar de ruído modulado - dB NPSpe	49,1	49,0	45,0 - 55,0
BMM - dB NPSpe	11,7	12,0	6,2 - 15,0

**Legenda:** dB NPSpe = decibel nível de pressão sonora; BMM = Benefício do Mascaramento Modulado; Q_25_ - Q_75_ = distância interquartílica.

Espectro preto = estímulo de fala /ba/; Faixa cinza (30 dB) = ruído estável fraco; Faixa cinza (65 dB) = ruído estável forte; Faixa cinza fragmentada (30, 65 dB) = ruído modulado; dB NPS (Decibel - Nível de Pressão Sonora)

Na Figura 3, pode-se inferir visualmente que o limiar na condição de ruído estável forte é acentuadamente elevado comparado com a condição de ruído modulado, com significância estatística no teste de Wilcoxon, p=0,003. Quanto ao BMM, foi possível observar que o intervalo de confiança foi de 7,7 a 15,7. Além disso, a diferença média entre os limiares eletrofisiológicos resultou em um BMM de 11,7 dB.

**Figura 3:** Limiar eletrofisiológico para o estímulo /ba/ em função do tipo de ruído

## DISCUSSÃO

O complexo das ondas corticais P1, N1 e P2 quando evocado por um estímulo de fala, revela respostas exógenas referentes às características acústicas do processamento sonoro^(14)^. No presente estudo, foi possível observar a presença do complexo cortical em todo os indivíduos avaliados, o que sugere que o estímulo de fala foi recebido adequadamente em nível do córtex auditivo.

Neste estudo, processo de investigação do BMM diante de potenciais auditivos corticais está relacionado a detecção do estímulo de fala em nível central, refletindo a atividade excitatória pós-sináptica, em nível do tálamo e córtex auditivo primário, além das áreas de associação^(15)^.

Analisar as latências e as amplitudes dos componentes corticais no processamento desses sinais complexos permite inferir sobre a influência do tempo na percepção do estímulo e sobre a magnitude da atividade cortical, respectivamente^(15)^.

No presente estudo observou-se que o maior tempo de detecção do estímulo deu-se na condição em que o /ba/ foi apresentado com o ruído estável forte, evidenciado pelas maiores latências presentes nos componentes corticais, exceto em P2 (Tabela 1). Este fato se deu devido ao maior efeito do mascaramento causado pelo ruído estável forte nas latências, com diferença significativa quando comparado à condição de ruído estável fraco (que tiveram menores latências), porém sem diferença estatística quando comparado ao ruído modulado, tendo um efeito mascarante similar nesse caso.

Achados semelhantes de aumento da latência nos potenciais corticais eliciados por estímulo de fala diante do ruído contínuo foram observados em adultos jovens, resultando em latências dos componentes P1, N1 e P2 progressivamente atrasadas diante desta condição ruído^(16)^. Este atraso das latências pode ser resultado do efeito mascarante na sincronização da atividade neural subjacente ao processamento auditivo, uma vez que o ruído altera o tempo de percepção do sistema auditivo.

No que se refere aos valores de amplitudes, enquanto resposta representante da magnitude da atividade cortical observou-se menor amplitude, ou seja, menor magnitude de resposta para os componentes P1, N1 e P2 quando o estímulo /ba/ foi apresentado com o ruído estável forte. Ao mesmo tempo em que maiores latências foram observadas no ruído modulado e estável fraco, com diferença significativa quando comparadas com a condição de ruído estável forte (Tabela 1).

Na Tabela 2 percebe-se que o efeito do mascaramento causado pelo ruído modulado nos valores de amplitude foi inferior ao efeito causado pelo ruído estável forte. Sendo assim, podemos considerar uma melhor magnitude da atividade cortical no processamento do estímulo verbal diante do ruído modulado.

Esse resultado pode ser explicado pelo fato de que modulações na intensidade do ruído causam diminuição da relação sinal-ruído e consequentemente aumentam a amplitude do estímulo evocado^(5)^. Desta forma, o aumento da amplitude do estímulo diante das modulações no ruído, resulta na diminuição da latência e aumento da magnitude das respostas do sistema auditivo, tendo em vista que tais medidas variam inversamente e diretamente com a amplitude do estímulo, respectivamente^(17)^.

Ao comparar potenciais corticais evocados pelo estímulo monaural /ba/ diante de ruído estável em forma de fala e ruído de banda larga modulado, com nível de sinal fixo de 65 dB NPSpe e diferentes tipos de relação sinal-ruído, pesquisadores observaram também uma diminuição sistemática na amplitude e aumento na latência na condição de ruído estável^(18)^.

Quanto à morfologia do complexo P1-N1-P2 nas diferentes condições de ruído, a pior configuração das ondas observada com ruído estável forte, quando comparado aos demais registros, pode ser explicada pela maior interferência vista nas medidas de latência e amplitude nesta condição.

Estudos também têm observado potenciais corticais robustos no ruído modulado quando comparado ao ruído contínuo, indicando um menor efeito mascarante do sinal sonoro no ruído modulado^(16,19)^.

Na pesquisa do limiar eletrofisiológico do presente estudo observou-se um menor limiar diante do ruído modulado com diferença estatística significativa em relação ao limiar no ruído estável forte (Tabela 3; Figura 3).

O maior limiar observado na condição do ruído estável forte pode indicar que os efeitos do mascaramento temporal foram mais robustos nessa condição.

Desta forma, houve um limiar médio de 11,7 dB inferior no ruído modulado, que corresponde com a literatura ao referir que o limiar de detecção de um sinal em presença de um mascaramento modulado é geralmente mais fraco do que em mascaramento constante/estável^(20)^.

Essa diferença no limiar entre as duas condições de mascaramento pode ser tomada como uma medida representante da habilidade de resolução temporal dos indivíduos, estando relacionada à integridade do processamento temporal ^(8)^.

Considerando a taxa de modulação no ruído de 25 Hz na presente pesquisa, estudos têm relatado que as taxas de modulações mais baixas, como por exemplo, entre 8 Hz e 20 Hz, produzem maiores espaços temporais de menor amplitude do ruído e consequentemente geram uma melhor magnitude do BMM^(5,6)^.

Ao investigar o BMM nos PEAC com estímulo de fala e relacioná-los à medidas comportamentais, foram obtidos limiares eletrofisiológicos médios de aproximadamente 69 dB para o ruído estável e 55,5 dB para o ruído modulado, encontrando um limiar médio de aproximadamente 13,5 dB inferior no mascaramento modulado, próximo ao encontrado no presente estudo. Diante deste resultado, os autores pontuaram que o limiar eletrofisiológico de fala foi mais baixo diante do mascaramento modulado quando comparado ao mascaramento estável e associaram o benefício da modulação à capacidade de processamento temporal do indivíduo^(8)^.

Em estudos com medidas comportamentais, onde a magnitude do BMM foi estudada em normouvintes, contatou-se uma variação entre 15 e 25 dB de melhora no reconhecimento de fala com a taxa modulação do ruído mascarante entre 8 e 20 Hz^(7)^.

Um estudo que observou a semelhança da magnitude do BMM entre os domínios eletrofisiológicos e comportamentais ressaltou que os testes eletrofisiológicos não são apenas informativos quanto aos mecanismos subjacentes, mas que também possuem o potencial de avaliar as habilidades de processamento temporal^(8)^. Além disso, relata que os limiares de PEAC evocados por estímulo verbal podem ser preditores confiáveis ​​de limiares de detecção de fala diante dos mascaramentos estáveis ​​e modulados.

No presente estudo, a menor diferença entre os limiares eletrofisiológicos, ou seja, o menor BMM foi de zero dB, não havendo resultado negativo deste fenômeno nos indivíduos estudados. A literatura aponta que o BMM favorece que o cérebro processe pistas acústicas da fala que não coincidem com as características do ruído mascarante^(2)^.

Esses achados são limitados predominantemente a jovens adultos sem perda auditiva, no entanto, servem de base para pesquisa com outras faixas etárias, além de populações com alterações específicas das habilidades auditivas, contribuindo para a pesquisa do BMM em PEAC. A análise do BMM pode ser realizada na população jovem, adulta e idosa, possibilitando a avaliação da habilidade de resolução temporal diante do fenômeno do mascaramento.

## CONCLUSÃO

Os achados do presente estudo demonstraram um menor efeito mascarante do ruído modulado nas medidas de amplitude dos componentes corticais P1, N1 e P2, podendo indicar sinais do BMM. O BMM de 11,7 dB representado pela diferença entre os limiares eletrofisiológico médio, aponta para uma menor interferência do mascaramento temporal na condição em que o estímulo /ba/ é apresentado com ruído modulado. Diante destes resultados e da contribuição para a pesquisa do BMM em PEAC, considera-se a utilização do ruído modulado como mascaramento mais eficiente nesta avaliação.
